# Human resource requirements for quality-assured electronic data capture of the tuberculosis case register

**DOI:** 10.1186/1756-0500-5-75

**Published:** 2012-01-27

**Authors:** Nguyen B Hoa, Chay Sokun, Chen Wei, Jens M Lauritsen, Hans L Rieder

**Affiliations:** 1 National Tuberculosis Programme Viet Nam, 463 Hoang Hoa Tham street, Badinh District, Hanoi, Viet Nam; 2 National Center for Tuberculosis and Leprosy Control, Phnom Penh, Cambodia; 3 National Center for Tuberculosis Control and Prevention, Beijing, China; 4 University of Southern Denmark, Institute of Public Health, Campusvej 55, DK-5230 Odense M, Denmark; 5 EpiData Association, Enghavevej 34, DK-5230 Odense, Denmark; 6 Department of Tuberculosis, International Union Against Tuberculosis and Lung Disease, 68 blvd Saint-Michel, 75006 Paris, France; 7 Institute of Social and Preventive Medicine, University of Zürich, Hirschengraben 84 CH-8001, Zürich, Switzerland

**Keywords:** Tuberculosis, Data entry time, Recording and reporting, Data quality, Surveillance, Electronic data

## Abstract

**Background:**

The tuberculosis case register is the data source for the reports submitted by basic management units to the national tuberculosis program. Our objective was to measure the data entry time required to complete and double-enter one record, and to estimate the time for the correction of errors in the captured information from tuberculosis case registers in Cambodia and Viet Nam. This should assist in quantifying the additional requirements in human resources for national programs moving towards electronic recording and reporting.

**Methods:**

Data from a representative sample of tuberculosis case registers from Cambodia and Viet Nam were double-entered and discordances resolved by rechecking the original case register. Computer-generated data entry time recorded the time elapsed between opening of a new record and saving it to disk.

**Results:**

The dataset comprised 22,732 double-entered records of 11,366 patients (37.1% from Cambodia and 62.9% from Viet Nam). The mean data entry times per record were 97.5 (95% CI: 96.2-98.8) and 66.2 (95% CI: 59.5-73.0) seconds with medians of 90 and 31 s respectively in Cambodia and in Viet Nam. The percentage of records with an error was 6.0% and 39.0% respectively in Cambodia and Viet Nam. Data entry time was inversely associated with error frequency. We estimate that approximately 118-person-hours were required to produce 1,000 validated records.

**Conclusions:**

This study quantifies differences between two countries for data entry time for the tuberculosis case register and frequencies of data entry errors and suggests that higher data entry speed is partially offset by requiring revisiting more records for corrections.

## Background

The recording and reporting system assists national programs in the management of tuberculosis. [1] The tuberculosis case register is the data source for the reports submitted by basic management units to the national tuberculosis program. All essential information on tuberculosis patients to produce the two types of quarterly reports on case finding and treatment outcome is recorded in the standard tuberculosis case register. [1]

The development of computerized implementation of tuberculosis recording and reporting to generate quarterly reports requires data entry into some kind of electronic data entry screen before various analyses or reports can be generated. [2] While analysis of an electronic data base is efficient and very flexible, data entry might be time-consuming and prone to errors which can only be appreciated and corrected with double-entry and validation. This requires additional human resources on top of the basic and indispensable requirement of a physical paper record.

Our objective was to measure the data entry time required to complete and double-enter one record, and to estimate the time for the correction of errors in the captured information from tuberculosis case registers in Cambodia and Viet Nam. This should assist in quantifying the additional requirements in human resources for national programs moving towards electronic recording and reporting.

## Methods

### Sampling

A representative sample of tuberculosis case registers was selected based on the list of all 140 management units in the public (governmental) sector in Cambodia and all 668 units in Viet Nam. A random selection of 30 units from each list was made by an independent collaborator. From each of these randomly selected management units, the Tuberculosis Case Register (henceforth the "case register") for two full calendar years was taken. The earliest permissible registration start was 1 January 2003 and the latest 31 December 2005.

### Study approval

Because of the retrospective, record-based nature of the study and the omission of capturing any patient names, each country decided to require only administrative approval. Ethical approval for the study was obtained from The Union Ethics Advisory Group.

### Data entry form and capture

The electronic data collection instrument was prepared with EpiData Entry (Version 3.1, freely available at http://www.epidata.dk). All variables recommended in the forms proposed by the International Union Against Tuberculosis and Lung Disease (The Union) [1] were captured, except for the name and address of the patient.

The data entry form was designed to be as efficient as possible, i.e. the length of each field was kept at the minimum required to allow automatic progress to the next field without hard carriage return after data entry in most instances. For instance, the case register has seven dates, (including the date registered, treatment start date, dates of bacteriologic examination at diagnosis, after 2(3), 5 and 7 months; and treatment result date). Each of these was entered as three separate variables for day, month, and year, with the computer calculating an exact date if all date components were known or an approximate date otherwise.

This approach served a triple purpose. Firstly, the year remained unchanged for about half of the records of the 2-year period and could thus be set to automatic repetition in the next record, requiring only confirmation with a single key stroke rather than re-entering the four digits. Secondly, automatically calculated exact dates (when all three date components were available) could be distinguished from automatically calculated approximated dates when fewer date components were available. Thirdly, it circumvented errors arising from style differences in writing dates among collaborating countries. Efficiency was further improved by using numeric coding coupled with labels, so that many variables required just a field length of 1 (e.g., sex of patient, intensive and continuation phase definitions, disease category and site, treatment outcome, etc.). Thus, while there were 37 variables in total that had to be entered, the total sum of field lengths was only 111, many of which (repeat fields) did only require a single key stroke despite a length of 4.

To allow validation of duplicate files a unique identifier was automatically composed for each record from the tuberculosis unit number (unique for one calendar year for a given unit), the registration year, the code of the treatment unit and the country.

In Viet Nam, data were double-entered by different and independent data entry persons. The two completed files from each tuberculosis treatment unit were sent to the country coordinator for comparison to identify any discordance between values for any variable for every pair of records. They were validated in a single step in EpiData Entry which compares each record of the first set on the unique identifier with the corresponding record of the second set. The generated report lists every record with at least one discordance, showing every field for that record with any discordance in its value. As any discordance arises as a result of a data entry error in either of the two records of a pair and it cannot be known in advance which of the sets has fewer errors, it was arbitrarily decided that a copy of the first set should always be the set for making corrections. This file was saved as the final dataset in which any error as ascertained by checking against the original physical record was corrected. As a result, three sets of files were available, allowing reproducing the data validation process. In Cambodia, the same system was used per protocol, but human resource constraints precluded different data entry teams and task switching within the team was done at their discretion. Validation of files remained the responsibility of the national coordinator.

In addition to study variables, data entry time was computer-generated and written to an access-blocked field, recording the time elapsed starting from the opening of a new record and completing entering the value for the last field, immediately before saving the record to disk. This field provided the basis to obtain an estimate for data entry cost but it also offered a control element whether data were truly double-entered. In every file there were isolated records that resulted in artificially long entry times if the data entry person was disturbed during entry before reaching the end of the record. The design was such that any later corrections in the record did not result in a change of the originally recorded data entry time.

### Data analysis

All analyses were done using EpiData Analysis (Version 2.2.1.171, freely available at http://www.epidata.dk). The 120 original sets and the 60 final datasets were separately combined into two respective sets for analysis, defining new variables and sub-sets of the dataset as required. Point estimates are shown with 95% confidence intervals for the mean or proportions where appropriate.

## Results

Each of the 30 pre-determined randomly selected case registers from each jurisdiction was successfully obtained. Each country collected information on patients registered during a 2-year period, Cambodia for the years 2003 and 2004, and Viet Nam for the years 2004 and 2005. There were 4,215 patients from Cambodia and 7,163 patients from Viet Nam. Each of the patient records from Cambodia had two records with the number of seconds recorded, thus 8,430 records were available. For the 7,163 patients from Viet Nam, only 14,302 records had the number of seconds recorded, 7,153 (10 missing) in the first, and 7,149 (14 missing) in the second set. Thus, a total of 22,732 records were available for the analysis of data entry time, 8,430 (37.1%) from Cambodia and 14,302 (62.9%) from Viet Nam.

### Data entry time

**Figure 1:**
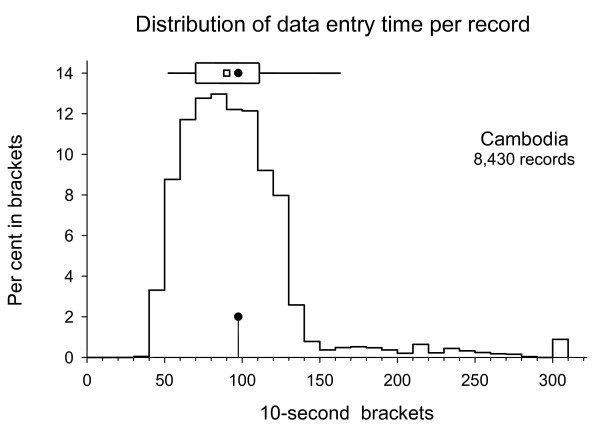
**Distribution of data entry times per record in Cambodia**. (The histogram shows the distribution in 10-second brackets (records requiring 300 or more seconds grouped into the last bracket). The top distribution shows the mean (filled circle), median (hollow square), 25% to 75% interquartile range (rectangle), and 5% to 95% range (horizontal line)).

**Figure 2:**
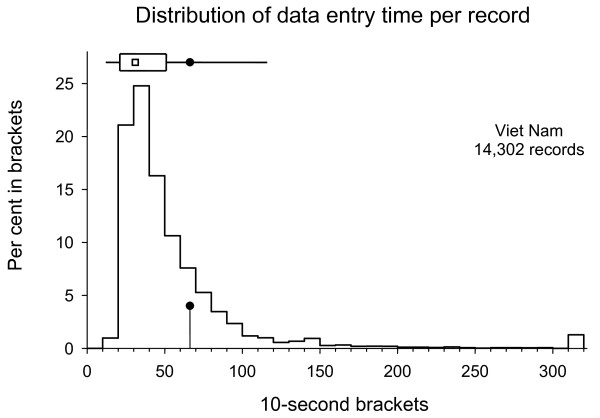
**Distribution of data entry times per record in Viet Nam**. (The histogram shows the distribution in 10-second brackets (records requiring 300 or more seconds grouped into the last bracket). The top distribution shows the mean (filled circle), median (hollow square), 25% to 75% interquartile range (rectangle), and 5% to 95% range (horizontal line)).

The results for data entry time are summarized in Table 1 and the distributions shown in Figures 1 and 2. The results demonstrate that the mean data entry time required in Cambodia was approximately 50% higher per record with 97.5 s than in Viet Nam with 66.2 s. The respective medians were 90 and 31 s. The distribution shows that very few records in Cambodia required fewer than 40 s, but in Viet Nam, the mode was actually at 30 to 40 s.

**Table 1:** Number of seconds per record of data entry tuberculosis case register in Cambodia and Viet Nam

Characteristic	Cambodia	Viet Nam	Total
Number of patients	4,215	7,163	11,378
Number of records with data entry time	8,430	14,302	22,732
**Data entry time (seconds)**			
Mean	97.5	66.2	77.8
95% confidence interval of mean	96.2 - 98.8	59.5 - 73.0	73.5 - 82.1
Median	90	31	52
**Error assessment**			
Number of common records	4,215	7,146	11,361
Number (%) of records with errors	251 (6.0)	2,787 (39.0)	3,038 (26.7)
Number of common fields *	240,255	426,950	667,205
Number (%) of fields with errors	433 (0.2)	8,920 (2.1)	9,353 (1.4)

*Due to small differences in the questionnaire design adapted to country-specific requirements, 57 fields per record were compared in Cambodia and 60 fields per record in Viet Nam (except for 58 fields in 5 units of the latter)

### Validating double data entry

**Figure 3:**
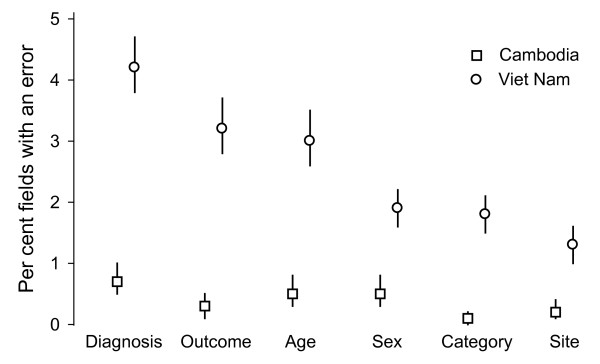
**Frequency of errors and 95% confidence interval in six key fields, Cambodia and Viet Nam**.

The measure of data entry quality is the proportion of fields with an error. (Figure 3) It must be noted, however, that a discordance for instance in a day field will show up as three errors as the automatically calculated exact and approximate dates will also be discordant. Thus correcting the error in the day in such an instance will automatically resolve all three discordances. The measure for the amount of work it will take to resolve all discordances will principally depend on the number of records, rather than the number of fields, that have to be revisited. The summary in Table 1 shows that in Cambodia only 6.0% of records, but in Viet Nam 39.0% of records showed any discordance between the pair of records. The proportion of records with any discordance depends on the number of fields that need to be entered per record as the probability of a data entry error in a given record increases with the number of fields.

The time required for checking the reported discordance in each record was not measured electronically. It had been done with a stopwatch in a pilot study of a tuberculosis laboratory register (Rieder H L, Gafner Zwahlen H, Spörry D, May 2006, unpublished). In that database with 1,620 common records, 105 (6.5%) had an error, a similar proportion as in Cambodia, but there were only 10 fields per record. The two data entry persons in the unpublished pilot study required 1.5 h to identify the discordant of records, check whether there was an error, and correct it if necessary. The total average data entry time for one set had been 9.47 h. In other words, the time required for record identification and correction required much more time than entering an entire new record. For the study here with the longer data entry forms, it appeared thus to be justified to assume that finding a record, checking the discordance(s), correcting them if necessary, and saving the revised record to disk would take the same time as entering a new record. The results of the calculations are summarized in Table 2.

**Table 2:** Data entry and correction times required for 1,000 validated final records of the tuberculosis case register, Cambodia and Viet Nam

Characteristic	Cambodia	Viet Nam	Total
Total number of records times two for double entry	8,430	14,302	22,732
Number of seconds per new record	97.5	66.2	77.8
Number of hours for all records	228.3	263.0	491.3
Number of records to check	251	2,787	3,038
Number of seconds for correction per record *	97.5	66.2	77.8
Total number of hours for correction	6.8	51.2	65.7
Total number of hours for validated records	235.1	314.2	556.9
Number of hours per 1,000 records	27.9	22.0	24.5
Number of hours per 1,000 validated records	55.8	43.9	49.0
Adjusted number of hours by 10-minute break hour per 1,000 records	66.9	52.7	58.8
Number of hours for 2 data entry persons per 1,000 records	133.9	105.5	117.6

* Identifying a record with a discordance, checking for the discordance, correcting an error if necessary, and saving the revised record to disk was assumed to take the same amount of time as entering a new record

As the computer-calculated number of seconds is pure data entry time, adjustment must be made for breaks to provide fair remuneration for work. For this study the recommendation was that 1 h should comprise 50 min work and 10 min break time. Furthermore, the data were always entered by a pair of workers. A total of 117.6 person-hours were thus required to obtain a final validated data set of 1,000 records, with a fairly small difference between Cambodia and Viet Nam.

In Viet Nam a professional team was employed for data entry, while in Cambodia the investigator took this task upon himself with colleagues and friends. It would appear that the professional team in Viet Nam worked much faster but also made considerably more errors that in themselves then required substantially more time for identifying and correcting. In contrast, the team in Cambodia worked slower but made substantially fewer errors, thus shortening considerably the time to make corrections. As a net result, there was only a small difference in the overall time required to produce a validated record.

## Discussion

In this study, we found that the median data entry time per record was three times larger in Cambodia than in Viet Nam. However, data entry time was inversely associated with error frequency: only six per cent of records in Cambodia had at least one data entry error as compared to almost 40% in Viet Nam.

We used freely available software for our study. Keeping up with proprietary software can accumulate to large costs that might be prohibitive for many low-income countries. The use of proprietary software will also prevent many researchers in such countries to carry out their research. Most importantly, for both affluent and low-income countries, proprietary software is mostly specified for analysis but not for quality-assured data entry. EpiData (EpiData Association, Denmark) and Epi Info (US Centers for Disease Control and Prevention) are the most important software packages that address both needs, they are free and allow efficient data entry and validation. [3]

With the spread of personal computers to intermediate and increasingly peripheral levels, an increasing number of countries has been initiating or is planning using electronic recording and reporting systems, referred to by the World Health Organization (WHO) as "e-R&R software" (http://www.who.int/tb/country/recording_reporting/en/index.html, accessed 1 December 2010). The advantages for analysis are apparent, yet any analysis is only as good as the quality of the input data.

At the above "TB e-Recording and Reporting Portal" of the WHO various systems are reviewed, most of which are based on proprietary software, and many depend on continuous and secure internet access. In the discussions of the Expert Group, the role of data quality is mentioned but no specific recommendations are made except for the suggestion that samples are checked for error frequency. Requirements of additional human resources for electronic data capture find no mentioning. In fact, an extensive evaluation of the experience with an electronic tuberculosis surveillance system in a low-income country, its pitfalls and successes alike, has, to our knowledge, only been published from one country. [2,4]

We conducted a country-wide representative study of tuberculosis registers in Cambodia, two provinces in China, and Viet Nam. [5] Because of the research nature of the study we required that the electronically captured data had to be an accurate reflection of the actual physical case registers. The thus chosen approach with double data entry and validation also provided an opportunity to quantify precisely the extent of errors made during data entry. Coupled with an inbuilt measurement of data entry time in Cambodia and Viet Nam, the dataset permitted determination of the cost in human resources to establish an as accurate as possible electronic dataset. Our analysis shows that it required 118 person-hours to obtain 1,000 validated patient records.

Importantly, the study demonstrates that the quality of primary data capture differed considerably between Cambodia and Viet Nam. This is likely the result of the actual differences in the arrangements made in the two countries: in Cambodia, the responsible researcher entered the data personally together with friends while in Viet Nam the task of data entry was outsourced but remained nevertheless under close supervision of the responsible researcher. The resulting differences are telling in that the quality of data entry was much superior where the researcher took personal responsibility than where the task was outsourced. Although the professional team worked considerably faster, this seeming advantage was nevertheless almost entirely lost by the longer time required to make all the necessary corrections. It is likely that in a routine setting supervision of data entry personnel with often little stake in quality-assured data is likely to be less tight than in this study setting. Because the extent of transcription errors will remain unpredictable and may vary greatly between settings, it would appear that quality assurance is an indispensable component of any research project specifically [6] and any routine electronic surveillance in general.

The system we employed was based on non-proprietary software and did not depend on continuous and high-speed internet access. Because EpiData software is text-based (http://www.epidata.dk), 1,000 records with 60 fields took less than 300 kilobytes, a file size that is easily transmitted as an E-mail attachment even with a slow and irregular internet connection.

This study has one main limitation. While the software allows measurement of cumulative time spent on each record, we did not foresee this in the design of our data entry form and recorded only the time needed for a first entry. We had thus to make the assumption (derived from external data) that finding a record, checking the discordance(s), correcting them if necessary, and saving the revised record to disk would take the same time as entering a new record.

While this study was done for research purposes, we believe that it has wider and generic implications for planning and budgeting in national tuberculosis programs. This is exemplified in this study, where the information of the recording time was necessary to budget payment for data entry persons based on an objective measure of delivery of validated records.

## Conclusions

Approximately 118 person-hours were required on average to produce 1,000 validated records of the tuberculosis case register. While professional data entry teams appear to work faster, they also make more errors. Our study seems to suggest that validating electronically captured data through double-entry and subsequent correction is an indispensable prerequisite for any epidemiologic research. The results demonstrate differences in quality of data entry, using identical data entry forms but different types of data entry teams. This suggests that our observations have important repercussions on electronic surveillance of tuberculosis in general if the objective is ensuring the quality of any subsequent analysis. The frequency of data entry errors varies greatly and cannot be known without actual data validation and analyses might thus be wrong. National tuberculosis programs need to pay special attention to this fact. They must take the necessary inbuilt precautionary measures in electronic database systems to reduce data entry errors. Barring double-entry and validation, a minimum requirement is ascertainment of error frequency in representative samples from the database.

## Abbreviations

e-R&R: electronic recording and reporting; TB: Tuberculosis; WHO: World Health Organization

## Competing interests

The authors declare that they have no competing interests.

## Authors' contributions

All the authors contributed to the design of the study. NBH, CS, CW were responsible for the implementing the study in the countries, data collection and data entry. HLR was responsible for the conception and overall supervision of the quality data collection, data entry and analysis. NBH, HLR, JML were responsible for data analysis. NBH and HLR wrote the first draft of the paper and all co-authors contributed to the writing of the final paper. All authors read and approved the final manuscript. HLR is guarantor of the paper.

## References

[B1] Enarson DA Rieder HL Arnadottir T Trébucq A International Union Against Tuberculosis and Lung Disease Management of tuberculosis. A guide for low income countries. (Fifth Edition) 2000 5 Paris: International Union Against Tuberculosis and Lung Disease 1 89 11263509

[B2] Alpers L Chrouser K Halabi S Moeti T Reingold A Binkin N Validation of the surveillance system for tuberculosis in Botswana Int J Tuberc Lung Dis 2000 4 737 743 10949325

[B3] Rieder HL Lauritsen JM Quality assurance of data: ensuring that numbers reflect operational definitions and contain real measurements. [State of the Art Series. Operational Research. Number 3 in the series] Int J Tuberc Lung Dis 2011 15 296 304 21333095

[B4] Vranken P Coulombier D Kenyon T Koosimile B Mavunga T Coggin W Use of a computerized tuberculosis register for automated generation of case finding, sputum conversion, and treatment outcome reports Int J Tuberc Lung Dis 2002 6 111 120 11931409

[B5] Hoa NB Chen W Chay S Lauritsen JM Rieder HL Completeness and consistency in recording information in the tuberculosis case register, Cambodia, China, and Viet Nam Int J Tuberc Lung Dis 2010 14 1303 1309 20843422

[B6] Rieder HL What knowledge did we gain through *The International Journal of Tuberculosis and Lung Disease *in 2008 on the epidemiology of tuberculosis? Int J Tuberc Lung Dis 2009 13 1219 1223 19793425

